# Electrophysiological Dynamics of Visual Speech Processing and the Role of Orofacial Effectors for Cross-Modal Predictions

**DOI:** 10.3389/fnhum.2020.538619

**Published:** 2020-10-27

**Authors:** Maëva Michon, Gonzalo Boncompte, Vladimir López

**Affiliations:** ^1^Laboratorio de Neurociencia Cognitiva y Evolutiva, Escuela de Medicina, Pontificia Universidad Católica de Chile, Santiago, Chile; ^2^Laboratorio de Neurociencia Cognitiva y Social, Facultad de Psicología, Universidad Diego Portales, Santiago, Chile; ^3^Laboratorio de Neurodinámicas de la Cognición, Escuela de Medicina, Pontificia Universidad Católica de Chile, Santiago, Chile; ^4^Laboratorio de Psicología Experimental, Escuela de Psicología, Pontificia Universidad Católica de Chile, Santiago, Chile

**Keywords:** orofacial movements, place of articulation, ERPs, viseme, articuleme, speech motor system, cross-modal prediction

## Abstract

The human brain generates predictions about future events. During face-to-face conversations, visemic information is used to predict upcoming auditory input. Recent studies suggest that the speech motor system plays a role in these cross-modal predictions, however, usually only audio-visual paradigms are employed. Here we tested whether speech sounds can be predicted on the basis of visemic information only, and to what extent interfering with orofacial articulatory effectors can affect these predictions. We registered EEG and employed N400 as an index of such predictions. Our results show that N400's amplitude was strongly modulated by visemic salience, coherent with cross-modal speech predictions. Additionally, N400 ceased to be evoked when syllables' visemes were presented backwards, suggesting that predictions occur only when the observed viseme matched an existing articuleme in the observer's speech motor system (i.e., the articulatory neural sequence required to produce a particular phoneme/viseme). Importantly, we found that interfering with the motor articulatory system strongly disrupted cross-modal predictions. We also observed a late P1000 that was evoked only for syllable-related visual stimuli, but whose amplitude was not modulated by interfering with the motor system. The present study provides further evidence of the importance of the speech production system for speech sounds predictions based on visemic information at the pre-lexical level. The implications of these results are discussed in the context of a hypothesized trimodal repertoire for speech, in which speech perception is conceived as a highly interactive process that involves not only your ears but also your eyes, lips and tongue.

## Introduction

Action-perception coupling has been the focus of extensive research in the field of cognitive neuroscience over the last decades. This research framework has led to a conception of the architecture of mind, that emphasizes the fact that behavior and neural dynamics are embedded in a body and situated in a context. It also reconsiders the importance of the agency and historicity of living organisms in shaping behavior and cognition (Maturana and Varela, 1987; Thompson and Cosmelli, 2011; Gomez-Marin and Ghazanfar, 2019). In line with those ontological principles, considerable efforts have been made to rethink traditional, modular accounts of speech and language (Tremblay and Dick, 2016; Duffau, 2018). Increasingly robust findings from the field of psycholinguistics (Glenberg and Gallese, 2012; Gambi and Pickering, 2013; Pickering and Garrod, 2013), computational neuroscience (Pulvermüller and Fadiga, 2010; Pulvermüller et al., 2016) and cognitive neuroscience (D'Ausilio et al., 2009; Peelle, 2019) suggest that ecological human communication is achieved by means of highly interactive multi-modal processes and feedforward predictions.

### Visemes-Phoneme Binding

While the association between speech sounds and articulatory representations of speech is well documented (Hickok and Poeppel, 2004, 2007; Okada et al., 2018), the interactions between visual and auditory forms of speech have only recently received considerable attention. Speech is not a purely auditory signal. A compelling illustration of the multisensorial integration of speech is the McGurk effect (McGurk and MacDonald, 1976). During ecological face-to-face interactions, perception of the speaker's orofacial articulatory movements offers critical complementary information for speech perception during infancy (Weikum et al., 2007; Lewkowicz and Hansen-Tift, 2012; Sebastián-Gallés et al., 2012; Tenenbaum et al., 2012), speech-in-noise perception (Sumby and Pollack, 1954; Ross et al., 2006), for non-native speech processing (Navarra and Soto-Faraco, 2005; Hirata and Kelly, 2010) and for people with hearing difficulties (Bernstein et al., 2000; Auer and Bernstein, 2007; Letourneau and Mitchell, 2013; Dole et al., 2017; Worster et al., 2017).

Imagine yourself, in a crowded party where the acoustic channel is overloaded by surrounding conversations, music and laughter. The perception of the articulatory movements of your friend's mouth would help you to cope with the challenging acoustic context, “perhaps by directing attentional resources to appropriate points in time when to-be-attended acoustic input is expected to arrive” (Golumbic et al., 2013, p. 1417). Visual information precedes auditory signals by 100-200 ms (Chandrasekaran et al., 2009). Thus, visual speech cues have the potential to serve a predictive function about the expected timing of upcoming auditory input (Arnal et al., 2009). For instance, if you see your friend opening her mouth, you would generate a prediction about her intention to initiate a conversation. This phenomenon, called predictive timing (Arnal and Giraud, 2012; van Wassenhove, 2013; Ten Oever et al., 2014), is especially relevant for turn-taking dynamics in human communication (Garrod and Pickering, 2015). In addition to providing temporal information about speech onset, visemes are particularly informative because the shape of the lips and/or the position of the tongue restrains the possible subsequent auditory input to a subset of possible phonemes. Seeing your friend pressuring her inferior and superior lips against each other would lead you to expect the upcoming sound to begin with a bilabial speech sound like /p/, /b/ or /m/. This phenomenon is known as predictive coding (van Wassenhove, 2007; Peelle and Sommers, 2015) and has been documented for both pre-lexical (Brunellière et al., 2013) and semantic (Økland et al., 2018) aspects of speech. Here, we will focus on cross-modal predictions in the context of speech perception, but it is important for the reader to be reminded that the neural feedforward processes taking place between auditory and visual modalities are not unique to speech, but rather rely on more domain-general dynamics of multisensory integration and error prediction (Kilner et al., 2007; Seth, 2013).

In electrophysiological studies, the effect of auditory facilitation, indexed by shorten latencies of the auditory evoked potential N1, is a well-documented consequence of cross-modal forward predictions [Shahin et al., 2018; also see Baart (2016) for a meta-analysis]. The N400, a component known for its responsiveness to semantic incongruence, has also been reported to be significantly enhanced in response to viseme-phoneme incongruence at the phonemic/syllabic level (Kaganovich et al., 2016; Kaganovich and Ancel, 2019). Interestingly, cross-modal facilitation and predictive coding have been shown to be modulated by visemic salience, with greater predictability for visemes with higher visual salience (van Wassenhove et al., 2005; Paris et al., 2013, 2017). Brunellière et al. (2013), for instance, reported an increase of late N400 component amplitude for visemes with highly salient visual cues (/p/) with respect to less salient visual cues (/k/).

It has been well-established by early fMRI studies that silent lip-reading produces an activation of auditory cortices (Sams et al., 1991; Calvert et al., 1997; Calvert and Campbell, 2003; Pekkola et al., 2005; Blank and von Kriegstein, 2013; Bernstein and Liebenthal, 2014). More recently, the analysis of oscillatory dynamics has consistently revealed that both auditory and visual speech perception induce neural entrainment at similar rhythms (Park et al., 2016, 2018; Assaneo and Poeppel, 2018; Poeppel and Assaneo, 2020). Importantly for the purpose of the current study, even in the absence of auditory input, the brain synthesizes the missing speech sounds based on visemic information. Silent lip-reading generates entrainment to the absent auditory speech at very slow frequencies (below 1Hz) in auditory cortices, even when participants do not know what the absent auditory signal should be (Bourguignon et al., 2018, 2020).

### *Articuleme*: The Smallest Distinctive Unit of Speech Motor Repertoire

Both speech sounds and their visual counterparts are physical outcomes of a sequence of coordinated articulatory movements of the vocal tract and orofacial effectors. We will refer to the articulatory neural patterns of activity that give rise to particular phonemes and visemes as *articulemes*. In this line, articulemes are conceptualized as partially invariant and language-specific patterns of neural and motor activity that, when instantiated, produce contrastive and meaningful linguistic information. This concept was first introduced by the Russian neuropsychologist Luria (1965, 1973) to label the specific articulatory patterns required to produce a phoneme (Ardila et al., 2020). Although this terminology has gone mostly unused for decades, we believe the notion of articuleme could reduce ambiguity in many current debates (see Michon et al., 2019). In fact, depending on disciplinary and theoretical background, the terminology used to refer to speech articulation varies (e.g., articulatory gesture, but also motor plan/program). We believe none of these terms is precise or clear enough to distinguish between the observed (visemes) and the produced (articuleme) language-specific orofacial gestures. Also, in light of recent evidence showing the relevance of the articulatory system in speech perception, we reintroduce the term *articuleme*. Here, we define it as the smallest unit of speech motor repertoire that can be isolated in the speech flux which produces meaningful and distinguishable elements of a given language.

Neuroimaging (Skipper et al., 2005; Pulvermüller et al., 2006; Correia et al., 2015; Archila-Meléndez et al., 2018) and TMS (Watkins et al., 2003; D'Ausilio et al., 2009; Sato et al., 2010; Swaminathan et al., 2013; Nuttall et al., 2018) studies have provided strong evidence of the participation of speech motor cortices during speech perception (but also see Stokes et al., 2019). Interestingly, using a variety of creative experimental procedures, a growing number of studies suggest that interfering with articulatory effectors negatively impacts speech perception. These sensorimotor influences on speech perception have been observed early in infancy: 6-month-old infants were unable to discriminate between non-native consonant contrasts when the relevant articulatory effector needed to produce the contrast was specifically restrained by a teething toy (Bruderer et al., 2015). Similarly, the ability of 7 years old children to recognize words by lipreading declined when they were holding a tongue depressor horizontally between their teeth (Bruderer et al., 2015). It has been suggested that language production system is required to elaborate predictions during speech perception (Pickering and Garrod, 2007). In this line, using fMRI Okada et al. (2018) showed that silent articulation of speech elicited greater activity than imagined speech in inferior frontal and premotor cortices and, although speech articulation was silent, in auditory cortex. The authors interpreted their results as an evidence of predictive coding, where activation of motor articulatory plans (here, articulemes) lead to predictions about the sensory consequences of those motor commands, which in turn serve to facilitate error monitoring and minimization. Martin et al. (2018) provided further evidence of the recruitment of language production systems during comprehension by demonstrating that the availability of the speech production system is necessary for generating lexico-semantic predictions, as indexed by greater amplitude of N400 when articulatory effectors were available vs. unavailable.

To summarize, the literature reviewed above suggests that visemes carry predictive information about forthcoming phonemes, with salient visemes producing stronger predictions of upcoming phonemes than less salient visemes. Strikingly, even in the absence of the auditory modality, the brain synthesizes the missing speech sounds on the basis of visemic content. Importantly, these cross-modal predictions between phonemic and visemic aspects of speech seem to depend on the speech motor system, and more specifically on the availability of the effectors required to generate particular speech sounds. This theory is often considered to be a modern and weaker version of Liberman's motor theory of speech perception (Liberman and Mattingly, 1985; Skipper et al., 2005; Massaro and Chen, 2008).

Although language perception and production have traditionally been studied as independent functional modules or “epicenters” (Tremblay and Dick, 2016), recent evidence points toward a highly interactive multimodal network that associates perceived orofacial movements with acoustic representation based on the motor sequences required to generate those movements. We recently proposed, based on this network, a trimodal repertoire of speech in which phonemes, visemes, and articulemes are bounded to achieve a more ecological, enactive and seamless perception of speech (Michon et al., 2019).

In contrast to the growing body of studies documenting the neuroanatomical circuits involved in audiovisual speech perception, the electrophysiological data available about the silent, visual processing of speech is still scarce. In the current study, two experiments were performed aiming to elucidate whether or not the linguistic content and the salience of visual speech cues modulates the electrophysiological responses elicited by perceiving silent orofacial movements and to what extent interfering with articulatory effectors can affect these responses.

## Methods

### Stimuli

The stimuli consisted in a set of 120 silent video clips displaying either no facial movement (1- still faces), one of a variety of orofacial movements (2- forward syllables, 3- backward syllables, 4- non-linguistic movements) or the movement of a purely geometrical shape (5- geometric). Videos were rendered into 1,080 ×1,920 pixel clips, lasting ~2 s (M = 2,052 ms and SD = 59 ms), with a frame rate of 29 frames per second. In the still faces condition (1), no mouth movements were produced (baseline). The forward syllable condition (2) contained videos of people producing consonant-vocal (CV) segments starting with phonemes that differed in their place of articulation (PoA) coarticulated with the vowel /a/. Three types of phonemes where included accordingly to their PoA: bilabial (/p/ or /b/), alveolar (/d/ or /t/) and velar (/g/ or /k/), which require lip, tongue-tip and tongue-back movements for their production, respectively. We chose these consonants because they have the common feature of being stop consonants, which means that they are articulated by closing the airway so as to impede the flow of air, then maintaining the airway closed thus generating a slight air pressure and finally generating the sound by opening the airway and releasing the airflow. Importantly, syllables with these three PoA have been reported to have different visual salience: syllables starting with bilabial movements are more salient than those starting with velar movements (van Wassenhove et al., 2007; Jesse and Massaro, 2010; Paris et al., 2013). In the backward syllables condition (3), the same videos described previously were reproduced backwards. Because of their particular motor sequence, CV formed with stop consonants cannot be pronounced backwards. In that sense, backward played syllables represent an ideal control condition because these kinds of articulatory movements are visually very similar to speech in their low-level features but at the same time they are not pronounceable, they are not present in our motor repertoire. In the non-linguistic condition (4), orofacial movements producing no audible sounds (e.g., tongue protrusion, lip-smacking) were presented. This condition was introduced to control the activity associated with the processing of orofacial movements that do not present linguistic meaning. Finally, to control for general movement perception, independently of its biological and face-related nature, a fifth condition was added where opening and closing movements of different geometrical figures (e.g., ovals, squares, triangles) were shown. These stimuli were generated and presented using PsychoPy (Peirce, 2009).

**Figure 1:**
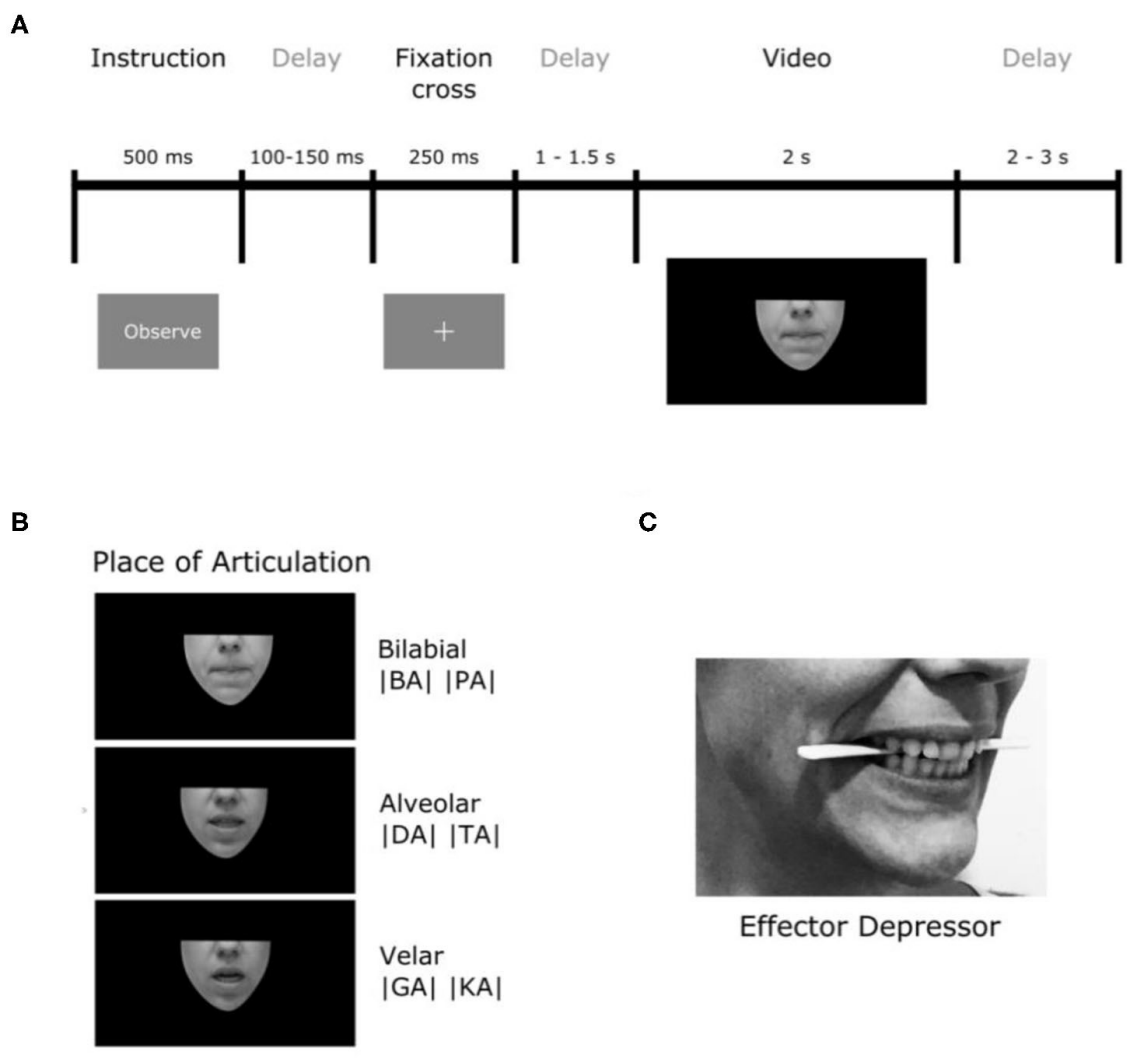
Experimental procedure. **(A)** Timeline description of trials. **(B)** Illustration of the places of articulation (PoA) of the syllables used. **(C)** Depiction of the position of the effector depressor in participants' mouths.

Importantly, all the videos were silently displayed (i.e., audio removed) and they only showed the lower part of the speaker's face (see Figure 1) in order to ensure that their eyes movements would not interfere. The software Adobe Premiere Pro CC 2017 (Adobe Systems) was used to edit the videos in a way that each began and ended with a still face (no mouth movements) or still geometrical shapes for condition 5 (sample videos for each condition are provided in the Supplementary Material).

### Participants

Thirty-two right-handed subjects (20 females) with normal or corrected-to-normal vision and hearing and without any history of psychiatric or neurological disorders performed the experiments. Participants' ages ranged from 18 to 36 years old (M = 22.8, SD = 4.2 years). The experimental protocol was approved by the Ethics Committee of Pontificia Universidad Católica de Chile. The procedure was explained to every participant and written informed consent was obtained from each one before the experiment began. Four participants were removed from the final analysis because of incomplete EEG recording or poor signal-to-noise ratio.

### Procedure

Participants sat at a distance of 70 cm from a computer screen and were asked to attentively observe or imitate the movements shown in the videos. The trial (see Figure 1A) started with a word lasting for 500 ms that indicate the instruction, either “Observe” (90% of the trials) or “Imitate” (10% of the trials). After 100 to 150 ms, a fixation cross appeared for 250 ms. In the observation condition, the video was displayed once, 1,000 to 1,500 ms after the white cross disappeared. In the imitation condition, the video was displayed a first time and participants were asked to attentively observe in order to co-imitate the orofacial gesture when the video was displayed for the second time. The onset of imitation was cued with a red fixation cross. After video offset, a new trial began within 2 to 3 s. The imitation condition was included as a sham task for the participants to maintain their attention on the stimuli and the experiment in general. No imitation instruction was given for purely geometric stimuli. The data from imitation trials were not analyzed.

To study the role of speech the motor system in speech perception, the very same experiment was repeated (Experiment 2) with the difference that participants were asked to hold a wood tongue depressor horizontally between their premolars, just behind incisors (see Figure 1C). This strategy, which produced an unnatural skin stretching of cheeks and lips, was introduced with the objective of generating a local motor perturbation of articulatory effectors of interest (e.g., lips). It is important to notice that the object used is called a tongue depressor because it is generally used by physicians in clinical settings to lower the tongue so they can observe the patient's throat. However, its use here was different, and aimed to interfere with speech articulations in the upper vocal tract. More precisely, due to the position of the tongue depressor, the motor perturbation acted more on lips and tongue-tip movements than on tongue-back movements. For this reason, we will refer to this object as “effector depressor.” In order to reduce muscle artifacts in the EEG signal, participants were asked not to squeeze their jaws, but to gently sustain the effector depressor between their premolars. For imitation trials, participants were asked to remove the depressor when the instruction “Imitate” appeared, so they could properly imitate.

Each of the 5 conditions (i.e., 1- still faces, 2- forward syllables, 3- backward syllables, 4- non-linguistic, and 5- geometric shape movements) consisted of 3 repetitions of 24 video-clips, leading to a total of 72 trials per condition (360 per experiment). The order of the conditions was pseudo-randomized across trials. For syllables conditions (2 and 3), an equal number of bilabial, alveolar, and velar syllables were used. The order of Experiment 1 and 2 was counterbalanced between participants.

### Electroencephalographic Recording Parameters

Electrophysiological activity was registered with a 64-channel EEG system (Biosemi ® ActiveTwo) with electrodes positioned according to the extended 10–20 international system. The signal was acquired with a sampling rate of 2,048 Hz and an online band-pass filter (0.1 to 100 Hz). Four external electrodes were used to monitor eye movements. Two of them were placed in the outer canthi of the eyes to record horizontal EOG and the other two were positioned above and below the right eye to record vertical EOG. Two additional external electrodes were placed bilaterally on the mastoids for re-referencing. Data pre-processing was performed using MATLAB (The Mathworks, Inc.) with EEGLAB (Delorme and Makeig, 2004) and ERPLAB toolboxes (Lopez-Calderon and Luck, 2014). Afterwards, the signal was down sampled to 512 Hz, re-referenced to mastoids and band-pass filtered between 0.1 and 40 Hz for ERP analysis. The 40 Hz low-pass filter ensured that muscle and 50 Hz AC current artifacts were removed. The EEG signal was then segmented into epochs from −500 to 1,500 ms respect to stimulus onset. Each epoch was visually inspected to reject large artifacts caused by head movements, electrode drifts or any amplitude changes exceeding ±100 μV. Then, Independent Component Analysis (ICA) decomposition was performed using the “binica” function (EEGLAB), the components typically associated with eye-blinking and the remaining artifacts were rejected using the MARA (“Multiple Artifact Rejection Algorithm”) plugin of EEGLAB.

### Statistical Analyses

The ERP components of interest for statistical analyses were N400 and a positivity around 1,000 ms (P1000). Using the ERP measurement tool in ERPLAB, mean amplitudes were calculated with respect to a 500 ms pre-stimulus baseline for the following selected time windows: N400 [475-525 ms] and P1000 [975-1025 ms]. These time windows were chosen based on the peak amplitude of each component. After mean amplitudes of those time windows were extracted for each condition and subject, data were analyzed using repeated measures ANOVAs. For comparisons relative to the PoA effect, 3-way (effector depressor × forward/backward × electrode) repeated measures ANOVAs were performed independently for the three types of syllables (bilabial, alveolar, and velar). When main effects were significant, simple main effect analysis was performed analyzing the difference of means between the levels of a single way of the ANOVA (e.g., comparison between bilabial | Forwards syllables | With v/s Without effector depressor). The reported *p*-values correspond to the significance of comparisons after Bonferroni corrections. For all statistical analyses involving more than two levels, the sphericity assumption was checked using Mauchly's test. In cases of a violation of the sphericity assumption, the Greenhouse-Geisser adjusted *p*-values were used to determine significance. Effect sizes are reported for all significant repeated-measures ANOVAs using the partial eta squared statistic ($\eta_{p}^{2}$). All statistical analyses were performed using JASP software (Version 0.12.2; JASP Team., 2020).

## Results

### N400

**Figure 2:**
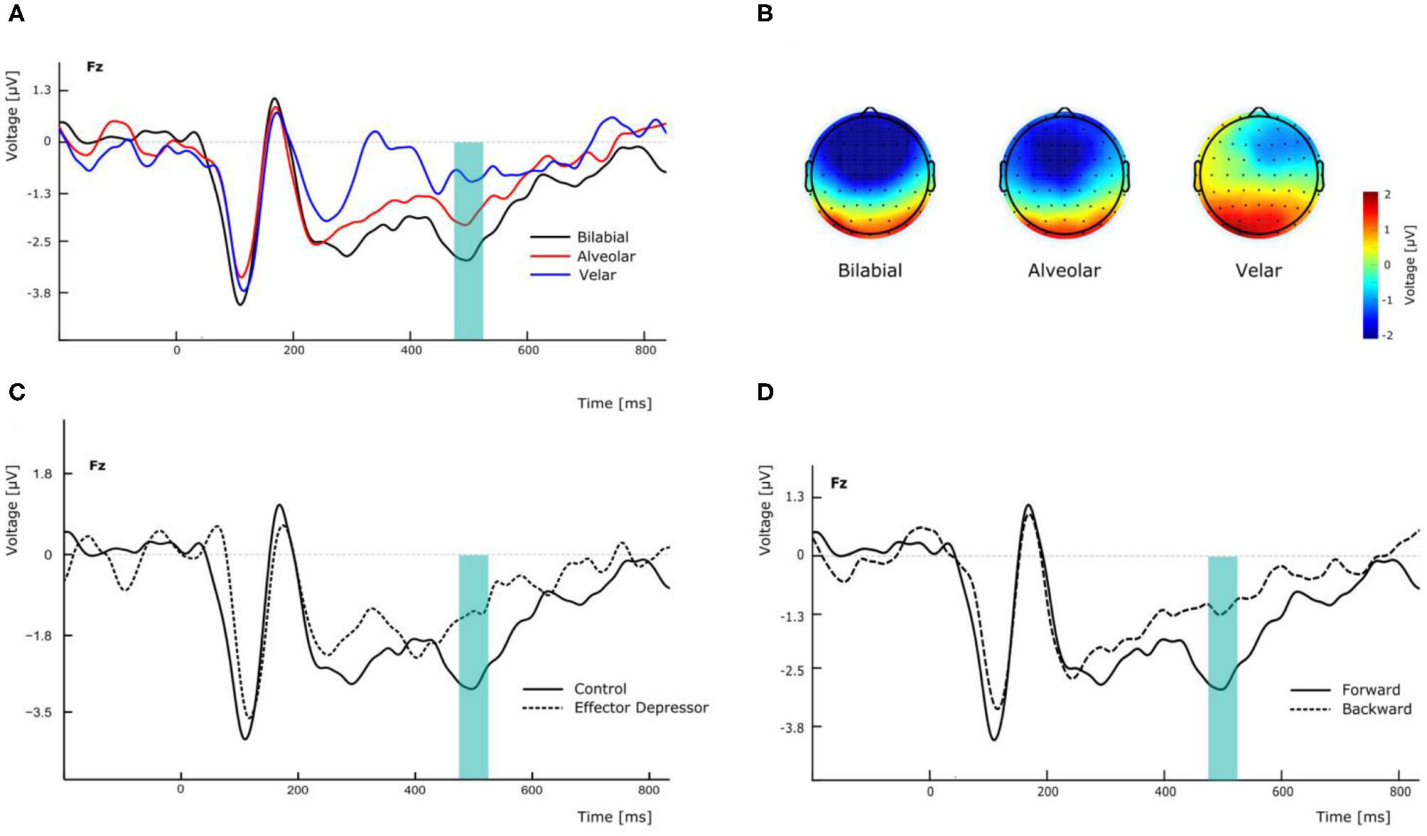
Effects of experimental manipulation on N400 component. **(A)** Effect of the different PoA of forward syllables in electrode Fz. **(B)** Topographical maps of the N400 according the PoA at the peak amplitude latency (500 ms). **(C)** Effect of effector depressor on the perception of forward bilabial syllables in electrode Fz. **(D)** Effect of forward versus backward syllables on the perception of bilabial syllables without effector depressor in electrode Fz.

N400 has been found to peak over fronto-parietal electrodes but also to be lateralized in similar linguistic settings (Kutas and Hillyard, 1980). In this line, to assess the effect that PoA could have in the amplitude of the N400 component, we conducted a repeated measures two-way ANOVA, with PoA and electrode (F3, Fz, and F4) as ways, and the N400 amplitude as the dependent variable, for forward syllables in Experiment 1. This analysis showed a significant main effect of PoA [*F*(2,54) = 4.576, *p* = 0.022, $\eta_{p}^{2}$ = 0.145; see Figure 2A]. More specifically, *post-hoc t*-tests comparisons (with Bonferroni correction) indicated that N400 amplitude was significantly greater for bilabial than for velar syllables across electrodes (mean difference = −2.037 μV, *p* = 0.012) whereas no significant differences were found between other PoAs. In this analysis, no significant main effect of electrode was found [*F*(2,54) = 0.657, *p* = 0.439, η^2^_*p*_= 0.024], however, the topological distribution of our N400 component is consistent with the literature (Figure 2B). Interestingly, the same analysis was run for Experiment 2, revealing no main effect of PoA [*F*(2,54) = 0.170, *p* = 0.844, η^2^_*p*_= 0.006] and no significant difference between bilabial and velar syllables (all corrected *p* > 0.05). This suggests that the effector depressor had an important impact on the elicitation of the N400 ERP component.

To better assess the differential effect of the effector depressor in bilabial CVs, we conducted a three-way repeated measure ANOVA for syllables with a bilabial PoA using Experiment, forward/backward and electrode as ways. This analysis revealed a significant interaction between Experiment (presence or absence of effector depressor) and forward/backward [*F*(1,27) = 10.219, *p* = 0.004, η^2^_*p*_= 0.275]. Simple main effect analysis showed that forward bilabial syllables elicited significantly greater N400 in the Experiment 1, in which participants freely observed the stimuli (control), compared to Experiment 2, where orofacial articulatory movements of the participants were restrained (effector depressor; Figure 2C). This effect was significant for all electrodes tested (F3, Fz, and F4; see Table 1), indicating the importance of the availability of motor effectors for the elicitation of N400. Importantly, this effect of the effector depressor was not observed for backward bilabial syllables. We then analyzed the simple main effects of bilabial syllables presented forward vs. backward. This analysis showed that forward syllables elicited greater N400 than backward syllables (Table 2). This effect was significant in all electrodes tested (F3, Fz, and F4 for Experiment 1, see Figure 2D) but were not significant for Experiment 2.

**Table 1:** Simple main effects of effector depressor on bilabial syllables.

**Experiment 1 vs. experiment 2**						
**FvsB**	**Electrode**	**Sum of squares**	**df**	**Mean square**	***F***	***p***
Forward	F3	23.582	1	23.582	4.233	0.049^*^
	Fz	35.970	1	35.970	5.335	0.029^*^
	F4	31.175	1	31.175	5.808	0.023^*^
Backward	F3	2.230	1	2.230	0.548	0.466
	Fz	2.195	1	2.195	0.405	0.530
	F4	4.277	1	4.277	1.043	3.316

* *p < 0.05*.

**Table 2:** Simple main effects of forward vs. backward displaying of bilabial syllables.

**Forward vs. backward**						
**Effector depressor**	**Electrode**	**Sum of squares**	**df**	**Mean square**	***F***	***p***
Experiment 1	F3	34.864	1	34.864	10.480	0.003^**^
	Fz	38.014	1	38.014	9.140	0.005^**^
	F4	39.363	1	39.363	8.393	0.007^**^
Experiment 2	F3	0.198	1	0.198	0.110	0.743
	Fz	1.726	1	1.726	0.640	0.431
	F4	1.898	1	1.898	0.607	0.443

** *p < 0.01*.

To further investigate the visemic modulation of N400, an additional two-way ANOVA was performed for electrode Fz in Experiment 1 with PoA and forward/backward as ways, eliciting a significant interaction [*F*(2,54) = 7.337, *p* = 0.002, η^2^_*p*_= 0.214]. More specifically, *post-hoc t*-test comparisons (with Bonferroni correction) indicated that N400 amplitude was significantly greater for forward bilabial CVs compared to backward bilabial CVs (mean difference = −1.648 μV, *p* = 0.005).

### P1000

**Figure 3:**
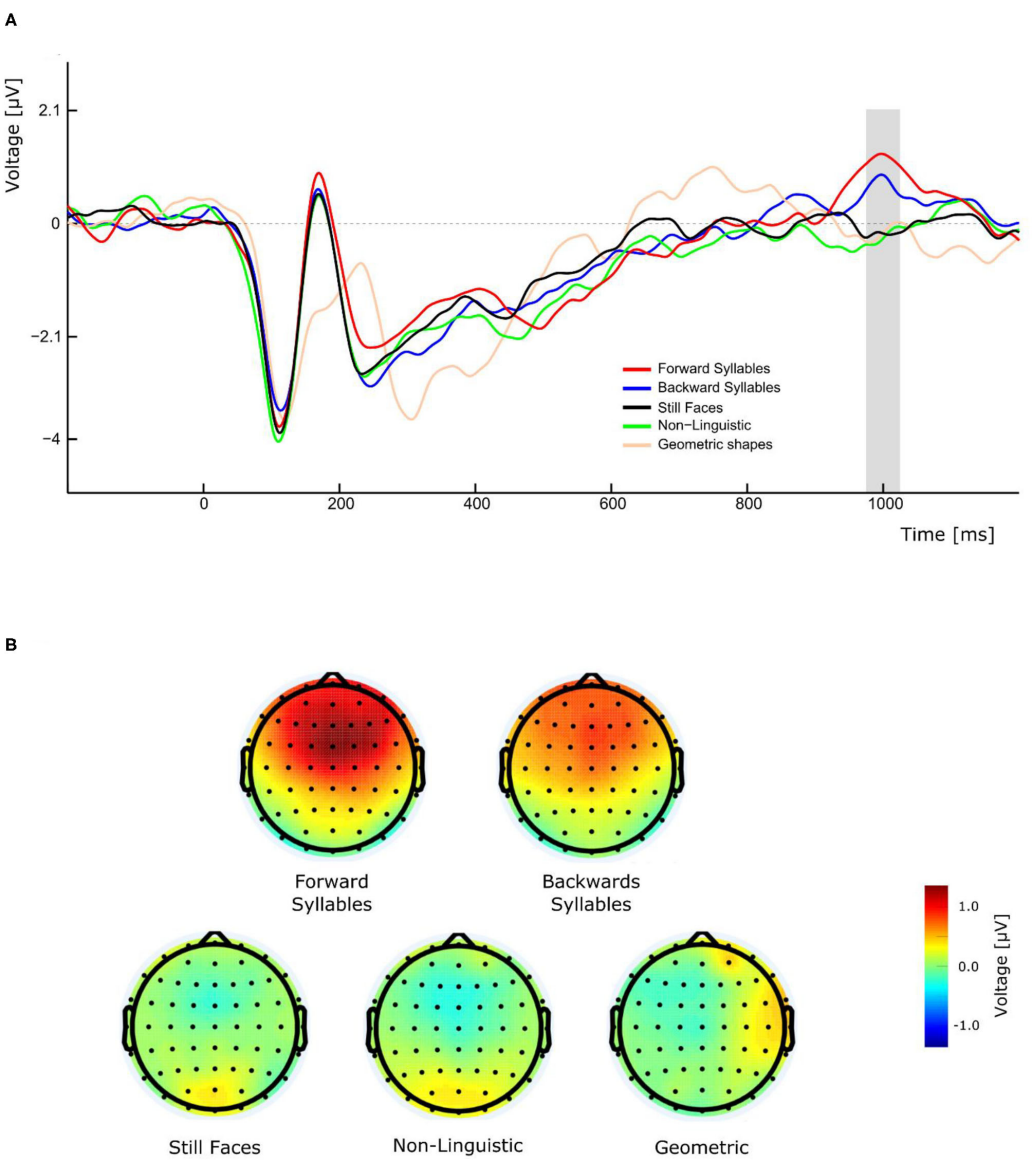
Effect of conditions on P1000 component in experiment 1. **(A)** The gray bar represents the time window in which significant differences in amplitude were found. **(B)** Forward and backward syllables (top maps) and non-syllabic conditions (bottom maps) showed different topographic representations.

As illustrated in Figure 3A, the perception of visual forward and backward syllables, independently of their PoA, produced a late positivity with a peak amplitude latency around 1,000 ms, which was absent in the control conditions (still Face, non-linguistic, and geometrical shape). A three-way repeated measures ANOVA (condition, Experiment, and electrode as ways) was conducted for the amplitude of this late positivity revealing a significant main effect of condition [*F*(4,108)= 9.684, *p* <0.001, η^2^_*p*_= 0.264]. *Post-hoc* pairwise *t*-tests comparisons (with Bonferroni correction) indicated that the amplitude was significantly greater for forward syllables and backward syllables respect to still faces (mean difference = −1.085 μV, *p* = 0.01 and mean difference = 0.873 μV, *p* = 0.009 for forward and backward syllables, respectively), to non-linguistic orofacial movements (mean difference = −1.040 μV, *p* = 0.001 and mean difference = 0.828, *p* = 0.004 for forward and backward syllables, respectively), and to geometrical shapes (mean difference = −1.025 μV, *p* = 0.014 and mean difference = 0.813 μV, *p* = 0.0019 for forward and backward syllables, respectively). Importantly, as illustrated in Figure 3B, a remarkable topographic difference was observed between syllables and non-syllabic stimuli, the former presenting a robust P1000 component over fronto-central regions.

## Discussion

The present study attempted to elucidate (1) whether or not the electrophysiological dynamics underlying perceptual processing of orofacial movements are modulated by the linguistic content and visemic salience of visual speech information, and (2) to what extent interfering with orofacial articulatory effectors can affect this process. In line with the reviewed literature, we formulated three rationales. First, if the missing speech sounds can be synthesized on the basis of visemic information only, different patterns of electrophysiological responses should be observed for visual speech cues vs. non-speech orofacial movements, since the former, but not the latter, have an associated auditory counterpart (e.g., backward syllables are not pronounceable and thus should lack an associated articuleme). The latter would advocate for cross-modal predictions during speech perception. Second, in line with previous results, within speech related movements, those with greater visual salience should show a greater effect of cross modal prediction (e.g., bilabial vs. velar syllables). Third, if it is the case that, additionally to visemic and phonemic dimensions of speech, articulatory motor patterns also play an important role in speech processing, the effect of cross-modal predictions should be disrupted by the interference of articulatory effectors introduced in Experiment 2. This would support the hypothesis of a trimodal repertoire of speech perception.

We observed that the N400 component was elicited for the syllable condition. In line with previous studies, we interpret the modulation of N400 amplitude as indexing predictive coding during speech perception. Since stimuli were silent, the increasing amplitude of N400 for increasingly salient visemes (Figure 2A) could reflect the error in prediction caused by the absence of the corresponding phonemes. Supporting this interpretation, Chennu et al. (2016) reported a negative deflection similar to the mismatch negativity in response to omitted sounds (i.e., the omission effect), indicating the presence of top-down attentional processes that strengthens the brain's prediction of future events (Chennu et al., 2016). Congruently, in our data the conditions in which no auditory counterpart was expected (e.g., still faces) did not elicited N400. This cross-modal facilitation and predictive coding have also been shown by other experiments manipulating visemic salience, which show that greater predictability is evoked by visemes with higher visual salience (van Wassenhove et al., 2005; Paris et al., 2013, 2017). Additionally, Bourguignon et al. (2018, 2020) have shown that silent lip-reading generates neural entrainment to an absent auditory speech, even when participants do not know what the absent auditory signal should be. In this context, our results support the link between visemic and phonemic dimensions of speech in terms of predictive coding during perception.

Additional evidence for this comes from the presence of N400 elicitation for forward syllables, which have an expected auditory counterpart, but not for backward syllables (Figure 2D). Importantly, we only employed stop syllables, which cannot be uttered backwards. Thus, backward syllables in our experiment lacked an auditory counterpart. This is consistent with the lack of N400 evoked in this condition. Consistently, it has been reported that, “during the processing of silently played lip movements, the visual cortex tracks the missing acoustic speech information when played forward as compared to backward” (Hauswald et al., 2018). Our results strongly suggest that the visual perception of backward CVs that cannot be produced do not generate expectations about an associated speech sound. This result provides preliminary support for our hypothesized trimodal repertoire for speech, since the perceived motor articulations do not belong to the participants' motor repertoire, they are not identifiable as articulemes and therefore, they lack audiovisual binding.

Directly in this line, another compelling argument supporting the trimodal network hypothesis is the effect of effector depressor on cross-modal speech prediction in our study. In Experiment 2, when orofacial effectors movements were restrained, the effect of cross-modal predictions was not observed (Figure 2C). Specifically, forward bilabial CVs ceased to elicit an N400 when the related motor effectors were disrupted by the effector depressor (Table 2). In a recent study (Martin et al., 2018), N400 amplitude was shown to increase in response to sentences containing unexpected target nouns compared to expected nouns, but the effect of expectation violation was not observable when speech production system was not available (i.e., when articulators were involved in a secondary task). The latter suggests that the availability of orofacial articulators is necessary for lexical prediction during lip-reading. The results of Experiment 2 support the idea of Martin et al. (2018) that speech effectors are important in generating speech predictions. However, since we used syllables and not words, the results of the current study further extend these results, suggesting that the motor involvement in speech predictions occur not only at the lexico-semantic level but also the pre-lexical level.

In addition to the expected N400, we also observed a late positive ERP component peaking around 1,000 ms, which was evoked only during the presentation of syllables (forward and backward) but not during any other condition. This effect is clearly illustrated by the topographical maps of the different conditions (Figure 3B). Importantly, this late positivity was not affected by the introduction of the effector depressor in Experiment 2. In the context of semantically unexpected sentence continuations, Van Petten and Luka (2012) reported that, following the N400, late positivities were elicited by low-plausible word completion. Similarly to the P1000 observed in the current study, these post-N400 positivities (PNP) are topographically distributed over frontal region and observed in time windows ranging from 600 to 1,200 ms after stimulus onset (deLong and Kutas, 2020). These anterior PNPs have recently been interpreted as a reintegration of the incorrectly predicted information in order to reach a new high-level interpretation (Kuperberg et al., 2020). In the context of our study, the elicitation of P1000 for speech related orofacial movements could be attributed to the reintegration and recuperation of the missing speech sounds. Even though this interpretation is more challenging to account for the presence of P1000 in response to backward CVs, it is possible that those stimuli have been re-interpreted as VCs (e.g., backward/ba/ being reinterpreted as/ab/). The latter, however, is more speculative and further studies are needed to clarify the functional significance of P1000 in this context. For instance, future research including a control experiment with full audio-visual stimuli could be helpful to disentangle this point.

To summarize, here we show that (1) electrophysiological dynamics underlying the perception of orofacial movements are modulated by the visemic salience of speech information, (2) when visemic salience is high (e.g., bilabial CVs) cross-modal prediction effects occur from visual to auditory modalities and (3) interfering with orofacial articulatory effectors can disrupt these feedforward processes. The current study, among an increasing body of evidence from the cognitive neuroscience literature on audiovisual speech processing and motor control of speech, points toward the necessity to rethink ecological speech perception beyond the auditory modality and include visual and motor systems in mechanistic explanations and neurobiological models of language.

## Data Availability Statement

The datasets generated for this study are available on request to the corresponding author.

## Ethics Statement

The studies involving human participants were reviewed and approved by Ethics Committee of Pontifical Catholic University of Chile, School of Psychology. The patients/participants provided their written informed consent to participate in this study. Written informed consent was obtained from the individual(s) for the publication of any potentially identifiable images or data included in this article.

## Author Contributions

VL and MM conceived and planned the experiments. MM carried out the experiments. GB and MM performed the data analyses. VL, GB, and MM contributed to the interpretation of the results. MM took the lead in writing the manuscript. Nevertheless, all authors provided critical feedback and helped shape the research, analysis, and manuscript.

## Conflict of Interest

The authors declare that the research was conducted in the absence of any commercial or financial relationships that could be construed as a potential conflict of interest.

## Supplementary Material

Supplementary Video 1 Still face.

Supplementary Video 2 Forward bilabial.

Supplementary Video 3 Forward alveolar.

Supplementary Video 4 Forward velar.

Supplementary Video 5 Backward bilabial.

Supplementary Video 6 Backward alveolar.

Supplementary Video 7 Backward velar.

Supplementary Video 8 Non-linguistic tongue.

Supplementary Video 9 Non-linguistic lips.

Supplementary Video 10 Geometrical shape.
